# Acanthocytosis and the c.680 A>G Mutation in the PANK2 Gene: A Study Enrolling a Cohort of PKAN Patients from the Dominican Republic

**DOI:** 10.1371/journal.pone.0125861

**Published:** 2015-04-27

**Authors:** Jasmin Schiessl-Weyer, Pedro Roa, Franco Laccone, Britta Kluge, Alexander Tichy, Euripedes De Almeida Ribeiro, Rainer Prohaska, Peter Stoeter, Claudia Siegl, Ulrich Salzer

**Affiliations:** 1 Department of Medical Biochemistry, Max F. Perutz Laboratories, Medical University of Vienna, Vienna, Austria; 2 Centro de Diagnostico Medicina Avanzada, Laboratorio y Telemedicina, Santo Domingo, República Dominicana; 3 Department of Medical Genetics, Medical University of Vienna, Vienna, Austria; 4 Platform Bioinformatics and Biostatistics, University of Veterinary Medicine, Vienna, Austria; 5 Department of Structural and Computational Biology, Max F. Perutz Laboratories, University of Vienna, Vienna, Austria; CINVESTAV-IPN, MEXICO

## Abstract

Pantothenate Kinase-Associated Neurodegeneration (PKAN) is a form of Neurodegeneration with Brain Iron Accumulation (NBIA) associated with mutations in the pantothenate kinase 2 gene (PANK2). Pantothenate kinases catalyze the rate-limiting step of coenzyme A synthesis and Pank2 is the only pantothenate kinase isoform in humans that is localized to mitochondria. Acanthocytosis, the occurrence of spiculated erythrocytes, is observed in about 10% of the PKAN patients. Therefore PKAN is also classified together with other rare neurodegenerative diseases like Chorea Acanthocytosis (ChAc) and McLeod syndrome (MLS) into the Neuroacanthocytosis (NA) syndromes. It has not been investigated yet whether acanthocytosis in PKAN is associated with a specific subset of Pank2 mutations. In this study, we analyzed acanthocytosis of a cohort of 25 PKAN patients from the Dominican Republic that are homozygous for the c.680 A>G mutation in the PANK2 gene as compared to control donors that are heterozygous or wild-type with respect to this mutation. 3D modeling of this mutation indicated that the replacement of a tyrosine by a cysteine at position 227 in Pank2 disrupts a polar interaction within the A domain of the enzyme. Mean acanthocyte count was elevated in the cohort of patients, however, acanthocytosis varied among the patients with nearly half of them showing high (>20%) or elevated acanthocytosis and the rest showing mild (6-10%) or no (<6%) acanthocytosis. Heterozygous control donors revealed a tendency to mild acanthocytosis. Based on the insight that Pank2 is a normal constituent of red blood cells and *de novo* biosynthesis of coenzyme A is likely to take place in the erythrocyte cytosol we propose a hypothetical model that accounts for the variability in the occurrence of acanthocytic cells in PKAN.

## Introduction

Pantothenate Kinase-Associated Neurodegeneration (PKAN) is known as the most frequent form of Neurodegeneration with Brain Iron Accumulation (NBIA) [1]. It is associated with mutations in the PANK2 gene [2]. Together with the other congenital disorders Chorea Acanthocytosis (ChAc), McLeod syndrome (MLS) and Huntington‘s Disease like-2 (HDL-2), PKAN is also classified into the clinical category of Neuroacanthocytosis (NA) syndromes indicating the coincidence of both neurological and hematological symptoms [3]. In contrast to ChAc and MLS where acanthocyosis is frequently associated with the disorders, the prevalence of apparent acanthocytosis (AC) in PKAN patients is reported to be only about 10% [2,4,5]. However, since acanthocytosis is not routinely assessed during diagnosis and therapy of PKAN, its prevalence among PKAN patients cannot be consistently derived from the available literature. Although the gene mutations are now known for all forms of NA, the molecular mechanisms that underlie the clinical symptoms are currently still under investigation [4,5].

The human genome comprises 4 PANK genes giving rise to various Pank proteins/isoforms that catalyze the first step of coenzyme A (CoA) biosynthesis. The four isoforms share a common, highly homologous, C-terminal domain that is responsible for the catalytic activity [6,7]. The N-termini of the Pank isoforms are variable and determine their subcellular localization [8]. Human Pank2 is the only isoform being localized to mitochondria [9]; moreover, Pank2 was recently also found to be localized to the nucleus [8]. Interestingly, as shown by comparative genomics, the mitochondrial targeting signal is limited to primates and is not present in mouse Pank2 [10]. This fact may be a reason for the mild phenotype of the PANK2 knockout mouse and points at the limitations of a mouse model to mimic molecular mechanisms in PKAN pathology [11].

PKAN typically presents in childhood with rapid progression over 10 years. Prominent neurological features include dystonia, speech difficulties, spasticity and choreoatheosis and in one third of the patients also cognitive impairment. Two courses of the disease can be distinguished: the classical early onset and the atypical late onset variety. Early onset PKAN starts before 6 years of age and shows rapid progression whereas in late onset PKAN, the average age of onset is 13–14 years and progression is slower [2]. Brain Magnetic Resonance Imaging (MRI) is the preferred examination technique in NBIA patients as specific patterns of iron accumulation along with clinical features may help in diagnostic processes and in monitoring of the disease progress [1,12,13]. T2 and T2 weighted MRI shows iron accumulations in the brain of PKAN patients especially in the globus pallidus and in the substantia nigra of the brain. In most of the of PKAN patients, a so-called "Eye of the Tiger" sign—a bright spot within the globus pallidus—can be detected, which probably represents the primary lesion and which is surrounded by an area of signal loss due to accumulation of iron [14].

The hematologic phenotype of acanthocytosis refers to an abnormal shape of the erythrocytes displaying unevenly distributed irregular spicules in contrast to the physiological biconcave shape of the red blood cells [15]. It is currently not known whether acanthocytosis coincides with or precedes the onset of neurological symptoms. The occurrence of acanthocytes has so far not been associated with specific clinical symptoms in NA patients. However, in vitro investigations of NA erythrocytes revealed various alterations of functional membrane properties, like reduction in drug-induced endovesiculation activity or impaired responses to lysophosphatidic acid [5]. Interestingly, in PKAN erythrocytes there was a coincidence of the occurrence of acanthocytes with these functional membrane alterations. Thereby, an “acanthocytic state” of the erythrocytes is suggested where alterations in interdependent membrane properties come together with an acanthocytic cell shape as observed in NA. Since the number of PKAN patients in that study was small and not homogenous with respect to the underlying mutation of the PANK2 gene, the question remained whether the PKAN-associated acanthocytic state of erythrocytes is caused by specific Pank2 mutations or rather by some Pank2-independent, yet unknown factor(s).

A cohort of PKAN patients was recently identified in a small region in the southwest of the Dominican Republic where the prevalence of PKAN is about 1000 times higher than normal [14]. In this study, blood samples of 25 PKAN patients and 64 control donors (mainly family members) were drawn and analyzed for the level of acanthocytosis and the response to a drug-induced endocytosis assay. The genotype of all donors with respect to the c.680 A>G mutation in the PANK2 gene was assessed. 3D modeling of the mutation suggested instability of the Pank2 protein. Moderate acanthocytosis was assessed in PKAN patients, however with large inter-individual differences. A hypothetical model for the occurrence of acanthocytosis in PKAN is proposed that accounts for this variability.

## Material and Methods

### Blood sample collection and fractionation

This study was approved by the ethics committee of the Medical University of Vienna (No. 1086/2012) and the national ethics committee from the Dominican Republic. Written informed consent was obtained from the participants before blood donation. Blood sample collection and transfer was organized in collaboration with the local medical support team. 10ml of blood (2 vials) were obtained by venipuncture from 89 donors, immediately cooled and transferred to Vienna. Whole blood samples were centrifuged for 15min at 1900 rcf, plasma and buffy coat were collected separately and stored at -20°C and -80°C, respectively. The packed red blood cells were mixed with an equal volume of polyvinylpyrrolidone (PVP) solution (30% in water, MW 10.000) and frozen as small drops in liquid nitrogen.

### Genetic and clinical evaluation of the patients

We tested for the c.680 A>G mutation of the PANK2 gene that had already been detected in the genetically confirmed patients [14]. DNA of the patients was extracted from buffy coat of the blood samples stored at -80°C. The coding exon 2 of the PANK2 gene (transcript: ENST00000316562) was amplified by PCR with the primers TTTGGAAACCCTAGCGTTTGAAAT and GATGGCCAACTCCTATCACTAGCG, and sequenced using a fluorescent automated sequencer (3130xl Genetic Analyzer; Applied Biosystems, Foster City, CA, USA) for the detection of the c.680 A>G mutation. The neurological examination and clinical status of the patients was done at the local hospital. MRI including at least one T2 weighted sequence was done on 24 of the 25 patients, either before or after taking the blood samples. One patient (DR61) was only diagnosed recently. Four of the patients that were part of the study meanwhile have deceased.

### Morphological analyses

Analyses of erythrocyte shape were performed upon fixation of the cells. To this end, 10 μl of whole blood were washed three times with PBS. Washed cells were mixed with 100 μl of fixing solution (1.5% paraformaldehyde, 0.1% glutaraldehyde in PBS), incubated at room temperature (RT) for 10 minutes, washed three times with PBS and suspended in 1ml PBS. For microscopy, 1 μl of fixed cell suspension was mixed (about 1.0x10^5^ cells) with 9 μl of PBS, placed on a microscope slide and allowed to settle at RT for 10 minutes. The supernatant was removed and the slide was left to air dry. The dried slide was fixed with pure methanol for 30 seconds and mounted with 80% glycerol, covered with a cover slip and sealed with nail polish. Morphological evaluations of at least 100 cells per sample were performed on Zeiss LSM Meta microscope using the 63x oil objective. We differentiated between two categories: normocytes (normal discocytic cell shape) and acanthocytes (including also other abnormal cell shapes). The counts (% of acanthocytes of total red cells) were grouped in four classes (normal (<6%), mild (6–10%), elevated (10–20%) and high (>20%).

### Drug induced endovesiculation

A flow cytometry-based assay for drug-induced endovesiculation was performed essentially as described in Siegl et al. [5]. Briefly, stored RBCs were thawed in pre-warmed PBS, washed three times with PBS containing 7.5 mM glucose, once in Hank's Buffered Salt Solution (HBSS; 0.137 M NaCl, 5.4 mM KCl, 0.25 mM Na2HPO4, 0.44 mM KH2PO4, 1.3 mM CaCl2, 1.0 mM MgSO4, 4.2 mM NaHCO3) containing 7.5 mM glucose and the volume was adjusted to a cell concentration of 2.5x10^9^ cells/ml. Fluorescein isothiocyanate (FITC) labelled dextran was added to the cells to give a final concentration of 10 mg/ml. Aliquots of 5x10^7^ cells were incubated for 30minutes at 37°C with primaquine bisphosphate at final concentrations of 0.75, 1.5, 2.0 and 3.0 mM, respectively. Finally, the cells were washed four times in PBS with 7.5 mM glucose and analyzed by flow cytometry (FACS Calibur, BD).

### Statistical analyses

All analyses were performed using IBM SPSS v20. The differences in measured parameters between groups (patients and controls) were analyzed using t-Tests for independent samples. Pearson’s correlation coefficient was used to determine correlation between AC (%) and primaquine concentration and some clinical parameters. The assumption of normal distribution was proven using Kolmogorof-Smirnov-Test. People were divided into two groups (< 10% AC, > = 10% AC). These groups were compared in frequency distribution of patients and controls using chi-square test. For all analyses a p-value of 5% (p < 0,05) was seen as significant.

## Results

### Clinical findings

17 patients had already been diagnosed with PKAN before. They are all homozygous for the c.680 A>G mutation in the PANK2 gene [14]. During the course of this study, we assessed the genetic status of all of the 89 blood donors. Seven patients with typical symptoms and MRI signatures were also genetically diagnosed to be homozygous for this specific PANK2 mutation. Moreover, one young and yet asymptomatic donor (DR61) turned out to be homozygous for this mutation. Two wild-type donors (DR70 and DR83) were diagnosed with sickle cell disease and were thus not further included in this study. Our sample set therefore comprised 25 genetically confirmed patients and 62 control donors, with 40 of them being heterozygous and 22 of them being negative for this PANK2 mutation. The mean age at onset of neurological symptoms was 10.8 ± 2.7 years (Table 1).

**Table 1:** List of patients and status of acanthocytosis.

ID	AC percent	age	onset
DR01	not rated	19	11
DR06	not rated	13	8
DR07	4.3%	25	10
DR11	17.2%	29	11
DR14	17.8%	11	10
DR15	22.3%	21	12
DR17	6.7%	25	10
DR20	19.0%	16	7
DR22	4.0%	31	12
DR29	20.2%	22	13
DR34	13.5%	18	9
DR39	3.4%	20	12
DR40	1.9%	33	11
DR43	14.2%	34	11
DR45	8.9%	29	9
DR48	7.7%	18	8
DR50	13.9%	13	12
DR54	3.7%	19	14
DR55	4.7%	21	11
DR57	14.7%	18	11
DR59	10.0%	14	14
DR61	3.7%	6	5
DR63	3.0%	14	7
DR79	9.0%	15	14
DR85	10.9%	19	18

ID denotes the internal identification code; AC percent is the percentage of acanthocytes within the blood sample; age and age at onset is given in years.

### Morphological evaluation of erythrocytes

**Fig 1:**
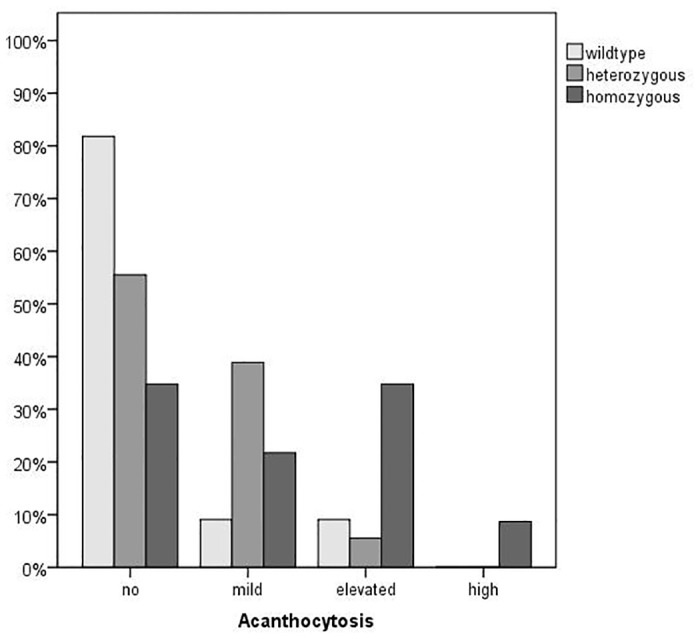
Degree of acanthocytosis in patients and control donors. Acanthocyte counts of donors that are heterozygous (n = 36), homozygous (n = 23) and wild-type (22) with respect to the c.680 A>G mutation in the PANK2 gene were microscopically assessed as described in the Materials and Methods section. The samples were grouped in four classes of acanthocytosis (normal (acanthocyte count <6% of total cells), mild (6–10%), elevated (10–20%) and high (>20%)) and the number of samples of each class is shown as relative percent of the total number in each subset of donors.

**Fig 2:**
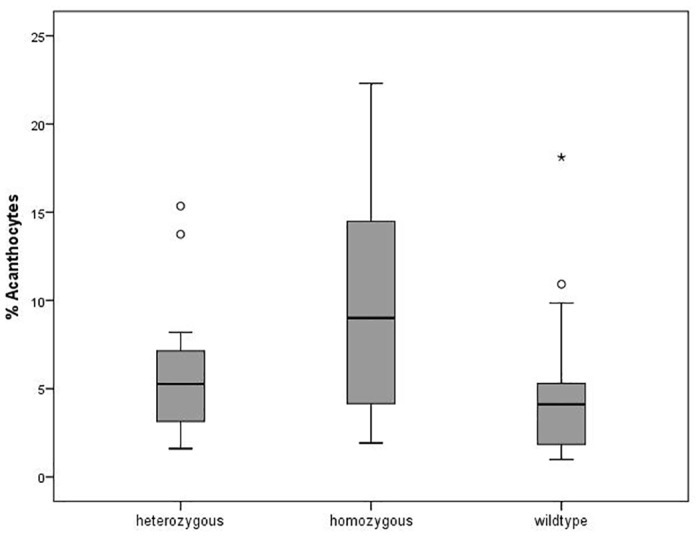
Distribution of acanthocytosis in the patient and control samples. The distribution of the amount of acanthocytes (in % of total cells) in the samples of donors that are heterozygous (n = 36), homozygous (n = 23) and wild-type (n = 22) with respect to the c.680 A>G mutation in the PANK2 gene is shown in a Box-Whiskers blot. The circles are *outliers*, and the asterisk is a *far outlier*.

The blood samples of two patients and four respective controls that were taken two days earlier showed an extreme endocytic behavior. Since prolonged storage might also have effects on cell morphology we disregarded these samples in the morphological ratings and the evaluation of endocytic properties (see below). Thus, for these analyses the sample set comprised 23 patients and 36 heterozygous and 22 wild-type control samples. Interestingly, acanthocytosis was a non-uniform feature among the group of patients. Whereas about 34% of the patients showed no abnormal acanthocyte count (< 6%) about 45% had an acanthocyte level above 10% and 2 patients had high acanthocyte counts (>20%). Expectedly, more than 80% of the wild-type control donors showed no acanthocytosis, however, 2 showed mild and 2 elevated acanthocytosis. Interestingly, 39% of the heterozygous donors (about 45%) showed mild acanthocytosis and 6% showed elevated acanthocytosis (Fig 1) indicating a tendency to moderately increased acanthocyte counts in carriers of the c.680 A>G mutation. This tendency to mild acanthocytosis in heterozygous donors as compared to wild-type donors is statistically significant as assessed by a chi square test (p = 0.048). The mean count of acanthocytes was 4.7% ± 4.0%, 5.5% ± 3.0% and 10.2% ± 6.3% for wild-type donors, heterozygous donors and patients, respectively. The difference between the value of the patients was significant compared to the value of the heterozygous (p = 0.004) and the wild-type donors (p = 0.013). A boxplot of the whole sample set is shown in Fig 2.

### Evaluation of endocytic properties

We have recently shown that blood samples of NA patients with a high acanthocyte count showed a significantly reduced response in a drug-induced endocytosis assay [5]. In order to assess this respective functional property of erythrocytes from the PKAN patients, we performed flow cytometry-based endocytosis assays for the same sample set as for the morphologic evaluation with 4 different concentrations of the endocytosis-inducing agent primaquine. Analyses of the results revealed that the mean value of endovesicle-positive cells is higher in wild-type and heterozygous donors than in patient samples for all 4 concentrations of primaquine (Table 2). This tendency corroborates our previous finding of a negative correlation between acanthocytosis and drug-induced endovesiculation. However, the differences were not statistically significant due to the narrow difference in the level of acanthocytosis between the patients and the control samples of our cohort.

**Table 2:** Primaquine-induced endovesiculation.

primaquine concentration	genotype	% endovesiclesmean ± stdev.	F(2,79)	p-value
0,75mM	homozygous	5,16 ± 3,29	2,25	0,112
	heterozygous	6,29 ± 3,50		
	wild-type	7,34 ± 3,54		
1,50mM	homozygous	20,07 ± 7,06	1,66	0,197
	heterozygous	21,18 ± 9,67		
	wild-type	24,51 ± 8,10		
2,00mM	homozygous	20,25 ± 9,23	1,02	0,364
	heterozygous	21,77 ± 9,50		
	wild-type	24,58 ± 12,46		
3,00mM	homozygous	56,90 ± 13,01	1,41	0,251
	heterozygous	60,75 ± 13,83		
	wild-type	63,23 ± 10,70		

Drug induced endovesiculation was assayed for blood samples of donors that are homozygous (n = 23), heterozygous (n = 36) and wild-type (n = 22) with respect to the c.680 A>G mutation in the PANK2 gene as described in the Material and Methods section using 0.75, 1.5, 2.0 and 3.0 mM primaquine. Mean values and the statistical significance of the difference between the three groups (assessed by an oneway ANOVA) are given.

### Molecular analysis of the c.680 A>G mutation

**Fig 3:**
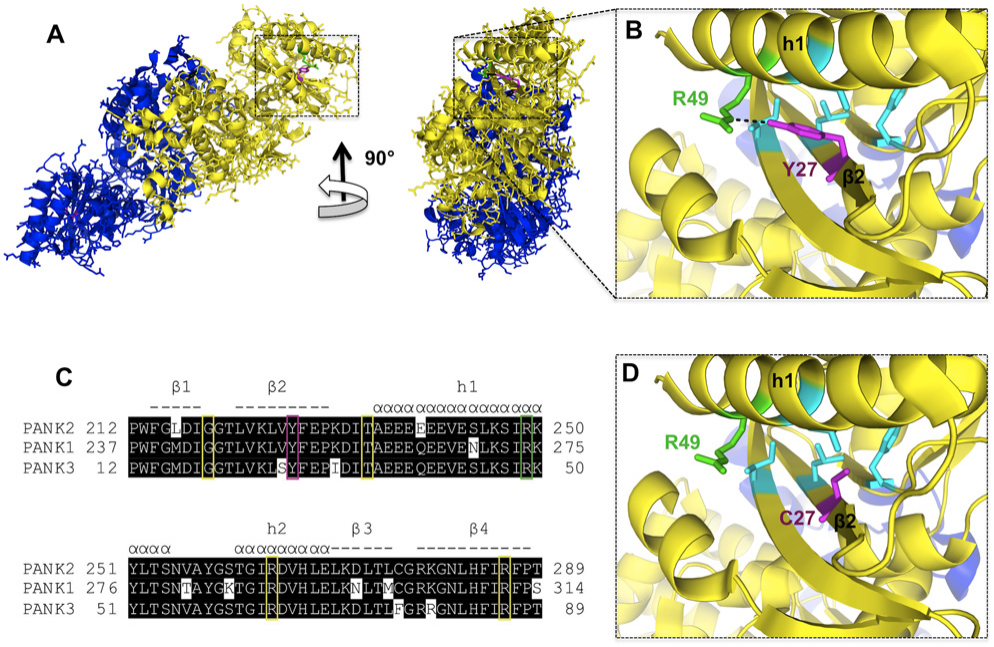
Mapping of the PKAN-linked Pank2 Y227C mutation onto the Pank3 structure und partial alignment of human Pank proteins. A) Pank3 dimer structure (PDB ID 2i7P) is shown in blue (chain A) and yellow (chain B), respectively. B) The magnification reveals a polar interaction (dashed line) in the WT Pank3 between Y27 (magenta, corresponding to Y227 in Pank2) and R49 (green, corresponding to R249 in Pank2). Side chains of F14, L25, L45 and I85 (cyan) are in close proximity to Y27 (less than 5 Ångström) constituting a hydrophobic environment around it. C) A partial multiple alignment of human Pank proteins, Pank2 (GI: 85838513), Pank1 (GI:23510400) and Pank3 (GI:119581908) is shown. Elements of secondary structure, helices (α) and β-strands (-) are indicated above and numbered accordingly. Known sites of PKAN missense mutations are boxed in yellow, the Pank2 Y227 site described here is boxed in magenta, the Pank2 R249 site which is in polar contact with Pank2 Y227 and is itself a known PKAN-linked Pank2 mutation is boxed in green. D) The magnification shows the effect of a Pank3 Y27C mutation (magenta) corresponding to the PKAN-linked Pank2 Y227C mutation disrupting the tyrosine-specific polar contact and local hydrophobic packing. PANK3 structure views and mutation Y27C were edited and modeled by PyMOL (http://www.pymol.org/). The contact map of Y27 was calculated using the Protein Interactions Calculator (PIC) server [24].

The cohort of PKAN patients is homozygous for a A>G missense mutation in exon 2 at the nucleotide position 680 of the transcript sequence (NCBI Reference Sequence: NM_153638.2) resulting in a cysteine instead of a tyrosine at position 227 of the Pank2 protein. Using the structures of the highly homologous Pank1 and Pank3 proteins as models, the conserved tyrosine at position 227 of the Pank2 sequence (corresponding to Y252 in Pank1 and Y27 in Pank3) can be mapped to a β-strand within the A-domain of the enzymes (Fig 3). This tyrosine (Y27 in Pank3) is in polar contact with an arginine (R49 in Pank3) of the adjacent h1 helix. Interestingly, the R249 of Pank2 corresponding to R49 of Pank3 is a known site of a missense mutation associated with PKAN. Biochemical analyses of Pank3 mutants corresponding to known disease mutants of Pank2 revealed that the R49P mutant still has full enzymatic activity but reduced stability *in vitro* [7]. These data suggest that the contact between Y227 and R249 in Pank2 could also be essential for the stability of the A-domain and replacement of one of these side chains may thereby lead to premature loss of activity *in vivo*. Hence the symptoms of the cohort of patients may arise from a reduction in but not a total loss of mitochondrial Pank2 activity.

## Discussion

The erythrocyte membrane is evolutionary adapted to respond to mechanical stress by immediate and reversible shape changes. This peculiar morphological flexibility is obtained by a complex interplay between the membrane constituents, various lipid species, a high amount of integral membrane proteins, and the network of the actin-spectrin cytoskeleton. Various congenital hemolytic anemias are known to be caused by mutations in erythrocyte cytoskeletal or membrane proteins and are associated with specific alterations in red cell shape and early removal of mature red cells from the circulation [16]. In contrast, in NA syndromes, the occurrence of acanthocytes is not associated with anemia or other hematological symptoms. Despite distinct primary causes, it is conceivable that a common factor/pathway influencing membrane shape homeostasis might be affected in all of the NA syndromes. Moreover, it is tempting to assume that the same factor/pathway is also active in neurons and its aberrant regulation is associated with neurodegeneration. Whereas VPS13A/chorein and the XK protein, the disease-causing proteins of ChAc and MLS, respectively, are known constituents of the red blood cell proteome, Pank2 was believed to be absent from erythrocytes since these cells eliminate mitochondria and all other cell organelles in the final maturation step. However, recent proteomic studies revealed that not only Pank2 but also all further enzymes of the coenzyme A biosynthetic pathway [17], namely phosphopantothenate-cysteine ligase, phosphopantothenoylcysteine decarboxylase and the bifunctional coenzyme A synthase, are normal constituents of the erythrocyte cytosol [18,19](_((((xxx. In contrast, other pantothenate kinases (Pank1, Pank3 and Pank4) that are known cytoplasmic proteins are seemingly absent from erythrocytes thereby implicating that erythrocyte de novo CoA synthesis is dependent on Pank2 activity. CoA as a carrier of acetyl or fatty acid moieties is a central player in a multitude of metabolic and signaling processes. For example, acetyl CoA has recently been shown to have a regulatory function by linking autophagic processes to the metabolic state [20] a process that might be of special importance during the maturation phase of erythrocytes. In this context it is interesting to note that reduced CoA levels and altered protein lysine acetylation patterns were found in a Drosophila model for PKAN [21]. Further, palmitoyl-CoA is the substrate for posttranslational modifications of target proteins with palmitic acid and reversible palmitoylation events are vital in mature erythrocytes involved in signaling processes (e.g. via Src kinases) and the organization of protein complexes at the erythrocyte plasma membrane [22]. Thus, Pank2 deficiency may result in aberrant lipid-based signaling processes and dysfunctional organization of protein complexes at the erythrocyte plasma membrane and thereby eventually lead to acanthocytic cell shape transformation.

In this study we show that (1) our specific cohort of PKAN patients has increased levels of acanthocytes compared to control donors (2) the degree of acanthocytosis varies among these patients despite of their identical Pank2 mutation and (3) donors being heterozygous with respect to this Pank2 mutation have an increased tendency to mild acanthocytosis. From (2) and (3), it can be concluded that the c.680 A>G mutation in the PANK2 gene alone is not sufficient to determine acanthocytic shape transformation in erythrocytes but some additional factor(s)/ condition(s) are necessary for acanthocytosis to occur. The following scenario of such co-founding factors/conditions is based on the hypothesis that reduced erythrocyte CoA levels are associated with acanthocytic shape transformation and on the assumption that the c.680 A>G mutation leads to an instability of the Pank2 protein. Since erythrocytes lack *de novo* protein synthesis, this instability would lead to a progressive depletion of Pank2 activity during the lifetime of the erythrocytes. However, chaperones likely counteract this loss of activity and variations in chaperone activity may therefore be one factor that could account for the non-uniform acanthocytic phenotype in PKAN patients. On the other hand, loss of Pank2 activity may not be critical as long as cytosolic CoA is not limited. This is corroborated by the biochemical finding that even low concentrations of CoA and several species of acyl CoA inhibit Pank2 activity thereby indicating that the requirement of Pank2 activity is coupled only to a drastic decrease in levels of cytosolic CoA (metabolites) [23]. The ability to maintain a super-critical cytosolic CoA level even in the absence of Pank2 activity may be another factor that could prevent acanthocyte formation in PKAN patients. This ability would presumably be influenced by variations in several metabolic determinants and possibly also by nutritional factors. We are aware that this model of acanthocytosis in the context of the c.680 A>G mutation is as yet speculative and has to be corroborated or dismissed by future studies on Pank2 activities and CoA levels in erythrocytes of these PKAN patients. However, if it holds true, this may be of general relevance for PKAN-associated acanthocytosis. In a recent study enrolling 11 PKAN patients that were heterogeneous with respect to the specific Pank2 mutation, we found mean percent acanthocyte counts of 30.9 ± 10.0 and 1.7 ± 1.4 for acanthocyte-positive (n = 6) and acanthocyte-negative (n = 5) PKAN blood samples, respectively [5]. In comparison, the mean level of acanthocytosis in the cohort of PKAN patients of this study is an intermediate value of 10.2 ± 6.3. Thus, a comprehensive model of PKAN-associated acanthocytosis will not only have to account for whether or not acanthocytes are present in the blood of patients but also for the different levels of acanthocytes in acanthocyte-positive blood samples.

The impact of various Pank2 mutations on the age of onset and disease progression in PKAN patients is not fully understood yet. However, the so-called “atypical” disease with late onset of symptoms (13.7 ± 5.9 years) has been shown to be associated with missense mutations in the PANK2 gene indicating that these patients may have residual Pank2 activity [2]. In line with our molecular analysis of the c.680 A>G PANK2 mutation, the delayed age of onset of 10.8 ± 2.7 years found in the patients from the Dominican Republic as compared to classical PKAN (3.4 ± 2.5 years^2^) is also indicative for residual Pank2 activity. Since pantothenate supplementation could possibly compensate for partial Pank2 deficiency [2], a respective nutritional initiative might ameliorate symptoms or at least retard disease progression.

Finally, future studies may take advantage of the yet unrecognized fact that Pank2 is a normal constituent of erythrocytes and CoA biosynthesis could principally take place in these cells. Thus, a small amount of blood donated by the patients will for example allow the biochemical characterization at the protein level of the various missense PANK2 mutations with respect to protein stability and eventual residual Pank2 activity and thereby serve as a model for the mitochondria-based defect of Pank2 that leads to neurodegeneration. It is likely that the readily accessible erythrocytes will turn out as a valuable complementary cellular system for future PKAN research.

## Supporting Information

S1 Table List of donors and status of acanthocytosis. “ID” denotes the internal identification code; “family” denotes the subset of families involved in this study; “status” indicates the degree of kinship within each family in relation to the patient, n.k. indicates that the status is not known, for donors of families without patients the kinship is not relevant and therefore the label “volunteer” is assigned; “genotype” is given with respect to the c.680 A>G mutation in the PANK2 gene; AC percent is the percentage of acanthocytes within the blood sample.
(PDF)
