# Supplementary material for: Acanthocytosis and the c.680 A>G Mutation in the PANK2 Gene: A Study Enrolling a Cohort of PKAN Patients from the Dominican Republic
Source: PLoS One. 2015 Apr 27;10(4):e0125861. doi: 10.1371/journal.pone.0125861 (PMC4411072; doi:10.1371/journal.pone.0125861)
Supplement: S1 Table — “ID” denotes the internal identification code; “family” denotes the subset of families involved in this study; “status” indicates the degree of kinship within each family in relation to the patient, n.k. indicates that the status is not known, for donors of families without patients the kinship is not relevant and therefore the label “volunteer” is assigned; “genotype” is given with respect to the c.680 A>G mutation in the PANK2 gene; AC percent is the percentage of acanthocytes within the blood sample. (PDF) [file pone.0125861.s001.pdf]

| ID | family | status | genotype | AC |
|---|---|---|---|---|
| DR01 | 26 | patient | homozygote | 3.6% |
| DR02 | 26 | mother | heterozygote | 6.7% |
| DR03 | 26 | aunt | heterozygote | 3.5% |
| DR04 | 24 | sister | heterozygote | 5.0% |
| DR05 | 24 | mother | heterozygote | 6.2% |
| DR06 | 24 | patient | homozygote | 7.2% |
| DR07 | 1 | patient | homozygote | 4.3% |
| DR08 | 1 | brother | heterozygote | 4.2% |
| DR09 | 1 | mother | heterozygote | 7.4% |
| DR10 | 1 | father | heterozygote | 2.7% |
| DR11 | 1 | patient | homozygote | 17.2% |
| DR12 | 4 | n.k. | wildtype | 4.6% |
| DR13 | 4 | mother | heterozygote | 5.0% |
| DR14 | 4 | patient | homozygote | 17.8% |
| DR15 | 4 | patient | homozygote | 22.3% |
| DR16 | 5 | daughter | heterozygote | 3.1% |
| DR17 | 5 | patient | homozygote | 6.7% |
| DR18 | 5 | mother | heterozygote | 3.6% |
| DR19 | 6A | sister A | wildtype | 1.1% |
| DR20 | 6A | patient A | homozygote | 19.0% |
| DR21 | 6B | father B | heterozygote | 7.7% |
| DR22 | 6B | patient B | homozygote | 4.0% |
| DR23 | 6B | sister B | wildtype | 3.9% |
| DR24 | 7 | n.k. | wildtype | 1.7% |
| DR25 | 7 | brother | heterozygote | 15.4% |
| DR26 | 7 | brother | heterozygote | 7.1% |
| DR27 | 7 | mother | heterozygote | 7.2% |
| DR28 | 7 | cousin | wildtype | 5.6% |
| DR29 | 7 | patient | homozygote | 20.2% |
| DR30 | 7 | cousin | wildtype | 1.8% |
| DR31 | 8 | brother | heterozygote | 2.2% |
| DR32 | 8 | brother | wildtype | 5.3% |
| DR33 | 8 | father | heterozygote | 3.2% |
| DR34 | 8 | patient | homozygote | 13.5% |
| DR35 | 8 | mother | heterozygote | 7.6% |
| DR36 | 9 | brother | heterozygote | 5.6% |
| DR37 | 9 | n.k. | heterozygote | 1.9% |
| DR38 | 9 | aunt | wildtype | 1.0% |
| DR39 | 9 | patient | homozygote | 3.4% |
| DR40 | 10 | patient | homozygote | 1.9% |
| DR41 | 10 | sister | heterozygote | 3.1% |
| DR42 | 11 | mother | heterozygote | 3.9% |
| DR43 | 11 | patient | homozygote | 14.2% |
| DR44 | 12 | father | heterozygote | 4.2% |
| DR45 | 12 | patient | homozygote | 8.9% |
| DR46 | 12 | n.k. | wildtype | 3.8% |
| DR47 | 13 | cousin | heterozygote | 7.1% |
| DR48 | 13 | patient | homozygote | 7.7% |
| DR49 | 13 | cousin | wildtype | 2.6% |
| DR50 | 13 | patient | homozygote | 13.9% |
| DR51 | 13 | cousin | wildtype | 2.0% |
| DR52 | 13 | mother | heterozygote | 3.1% |
| DR53 | 15 | mother | heterozygote | 4.3% |
| DR54 | 15 | patient | homozygote | 3.7% |
| DR55 | 15 | patient | homozygote | 4.7% |
| DR56 | 15 | father | heterozygote | 5.2% |
| DR57 | 21 | patient | homozygote | 14.7% |
| DR58 | 21 | mother | heterozygote | 3.8% |

| | | | | |
|---|---|---|---|---|
| DR59 | 21 | patient | homozygote | 10.0% |
| DR60 | 21 | n.k. | heterozygote | 13.8% |
| DR61 | 25 | patient | homozygote | 3.7% |
| DR62 | 25 | sister | heterozygote | 1.6% |
| DR63 | 25 | patient | homozygote | 3.0% |
| DR64 | 25 | brother | heterozygote | 6.1% |
| DR65 | 25 | mother | heterozygote | 2.5% |
| DR66 | 28 | volunteer | heterozygote | 7.9% |
| DR67 | 28 | volunteer | wildtype | 1.6% |
| DR68 | 28 | volunteer | wildtype | 4.3% |
| DR69 | 29 | volunteer | wildtype | 18.1% |
| DR71 | 30 | volunteer | wildtype | 9.9 % |
| DR72 | 30 | volunteer | heterozygote | 6.0% |
| DR73 | 30 | volunteer | heterozygote | 6.3% |
| DR74 | 30 | volunteer | heterozygote | 6.3% |
| DR75 | 31 | volunteer | wildtype | 10.9% |
| DR76 | 31 | volunteer | wildtype | 4.2% |
| DR77 | 31 | volunteer | wildtype | 4.7% |
| DR78 | 31 | volunteer | wildtype | 4.4% |
| DR79 | 32 | patient | homozygote | 9.0% |
| DR80 | 32 | sister | heterozygote | 5.3% |
| DR81 | 32 | sister | heterozygote | 8.2% |
| DR82 | 32 | mother | heterozygote | 8.1% |
| DR84 | 33 | cousin | wildtype | 4.0% |
| DR85 | 33 | patient | homozygote | 10.9% |
| DR86 | 34 | volunteer | wildtype | 6.2% |
| DR87 | 34 | volunteer | wildtype | 1.3% |
| DR88 | 35 | volunteer | heterozygote | 2.2% |
| DR89 | 35 | volunteer | heterozygote | 6.7% |
